# Framework for the Development of Affective and Smart Manufacturing Systems Using Sensorised Surrogate Models

**DOI:** 10.3390/s21072274

**Published:** 2021-03-24

**Authors:** María Jesús Ávila-Gutiérrez, Francisco Aguayo-González, Juan Ramón Lama-Ruiz

**Affiliations:** Design Engineering Department, Polytechnic School, University of Seville, 41011 Seville, Spain; faguayo@us.es (F.A.-G.); jrlama@us.es (J.R.L.-R.)

**Keywords:** affective workplace, activity theory (AT), variety law (VL), occupational environment, industry 4.0, intellectual disability, key enabling technology (KET), operator 4.0

## Abstract

Human Factor strategy and management have been affected by the incorporation of Key Enabling Technologies (KETs) of industry 4.0, whereby operator 4.0 has been configured to address the wide variety of cooperative activities and to support skills that operate in VUCA (volatile, uncertain, complex, and ambiguous) environments under the interaction with ubiquitous interfaces on real and virtual hybrid environments of cyber-physical systems. Current human Competences-Capacities that are supported by the technological enablers could result in a radically disempowered human factor. This means that in the processes of optimization and improvement of manufacturing systems from industry 4.0 to industry 5.0, it would be necessary to establish strategies for the empowerment of the human factor, which constitute symbiotic and co-evolutionary socio-technical systems through talent, sustainability, and innovation. This paper establishes a new framework for the design and development of occupational environments 5.0 for the inclusion of singularized operators 4.0, such as individuals with special capacities and talents. A case study for workers and their inclusion in employment is proposed. This model integrates intelligent and inclusive digital solutions in the current workspaces of organizations under digital transformation.

## 1. Introduction

Industry 4.0 allows new types of interactions between human factors, technology, and machines. These interactions are generating a new workforce that has significant repercussions on the nature of work [1]. The integration of workers into an industry 4.0 system that consists of different competences, capacities, educational levels, and cultural backgrounds presents a significant challenge [2]. The increase in the degree of digitalization in industrial plants involves an increased complexity of the daily tasks of human operators. Today’s workers are required to be highly flexible and to demonstrate adaptability in a dynamic work environment [3]. 

Human-centric manufacturing has constituted a primary topic for most previous manufacturing systems, such as lean production, resource efficiency, sustainable development, smart manufacturing, and advanced manufacturing [4,5]. Operator 4.0 is understood as an intelligent operator who performs cooperative and assisted work with machines through advanced technologies and automation of work systems. Operator 4.0 typology is useful in increasing the understanding of the future roles of humans and machines in the factories of human cyber-physical systems. Operator 4.0 vision includes smart factories of the future that are perfectly suited for workers with a variety of skills, capabilities, and preferences [6]. Human factors will have to prove their relevance in a digital age [7], because tacit knowledge regarding an individual’s experience cannot be transferred to robots and computers [8].

In this context, the term Industry 4.0 comprises a variety of technologies to enable the development of a digital and automated manufacturing environment. Industry 4.0 is considered the road to digitization in future manufacturing, and it would not render humans obsolete in industrial production [9]. 

After Industry 4.0, Industry 5.0 is defined as “Society 5.0”, a human-centric society that balances economic progress with the resolution of social problems by a system that closely integrates cyberspace and physical space [10]. In other words, Society 5.0 is a model for communicating a vision of a future society to industry and the general public. This model is the culmination of numerous discussions among experts from various fields. It was also based on research into the history of technology and social development [11]. The phenomenon visualizes a forward-looking society without information stagnation. When comparing Industry 4.0 with Society 5.0, similar technologies are used, such as artificial intelligence, cyber-physical systems, big data, the internet of things, robots, augmented reality, and the cloud. While Industry 4.0 is effective in a limited scope, as its area of practice involves industry, Society 5.0 chooses the whole of society, including industry, as an area of practice for itself. Furthermore, Society 5.0 deals with many social aspects, such as health, poverty, prosperity, easy access to jobs, and gender equality, whereas Industry 4.0 focuses mainly on cost reduction and more efficient production techniques in industry [12]. 

One of the objectives of Industry 5.0 is to improve the focus on the human factor. Empowering the worker is based on adapting the factory shop floor to the skills, capabilities, and needs of the worker in order to understand and develop his/her competence. Engagement of the work community is based on tools with which the workers can participate in designing their work and training, and can share their knowledge with each other [13]. Successful manufacturing systems in Industry 5.0 will be possible if the socio-technical systems are configured with the human factor, and are thereby rendered capable of adapting and enhancing reasoning, awareness, rationality, creativity, complex problem-solving, social relationships, and emotionality by showing features of human intelligence. Industry 5.0 constitutes an argument in favor of finding a place for humans (and humanness) in the future digital economy [7].

The natural provider for the configuration and implementation of occupational environments for a variety of workers in industry 4.0 involves the existence of frameworks, methodologies, and tools to carry out the process of design and development of smart systems, products, workplaces, and occupational environments. There are currently general design frameworks available, such as inclusive design [14], participatory design [15], occupational ergonomics (as a scientific discipline that studies the relationships between a person, the activity she/he performs, and the elements of the system in which she/he is immersed) [16], and collaborative design and computation [17]. 

The ability of today’s society to exploit and systematically develop existing innovation potential increasingly determines its future sustainability. An underlying understanding of innovation is crucial for the full development of technological potentials and their integration into sustainable development processes [18]. From the potential of the digital enablers of industry 4.0, workplaces can be conceived with criteria of sustainability, evolutionary stability, dynamism, and minimum complexity [19,20,21]. In order to control and enable smart integration of the worker into the workplace environment and for this worker to execute the work in an integrated way, a wide variety of technologies are available. Key enabling technologies (KETs) offer the technologies that are expected to enhance the competitiveness of the European small and medium-sized enterprises (SMEs) and to significantly contribute to solving the societal and environmental challenges [22]. 

Based on analyzing the possible reasons for transferring the knowledge of the workers to the technology, one of the main problems that arises involves the lack of frameworks, methodologies, and tools to develop solutions whose results ensure real and efficient integration of the human factor into dynamic environments. This paper therefore has various objectives: (1) detection of the potential of integration and co-evolution according to their variety and expectations that can be key to achieving the objectives of integrating the human factor from its experience, cognitive, and affective variety; (2) successful integration of the human factor at the level of processes, products, and business models that guarantees the system–environment–human adaptation; and (3) synchronization of the development of talent with the introduction of digital technologies, thereby the effectiveness and efficiency of the productive processes, and satisfying the real expectations of the people within the configuration of inclusive work environments and connectivity.

This paper aims to provide answers to the following research questions (RQs): (RQ I) Is it possible to establish a model for the inclusion of workers into the labor market in which they will benefit from the development of an independent life and be considered an active part of society in all phases of their lifecycle? (RQ II) Is there a generic theoretical framework or working methodology that helps in the decision-making process regarding the selection of functional requirements and design parameters of workplaces and occupational environments that encompasses the wide variety of needs, the complexity of signs and behaviors, and the modern digital world? (RQ III) How can the digital transformation potential of industry 4.0 and an innovative model from new conceptual frameworks be articulated for accessibility and social inclusion of workers and help regulate emotions in the workplace? Along these lines, the opportunity to develop a design model and methodology is detected that allows companies to adapt their workplace and improve their sustainability, and to promote their social implication. The key is to find an intervention model that benefits both parties, and all stakeholders in general, while promoting the employment of special workers and maintaining levels of productivity [23]. 

The manuscript has been structured in accordance with the generic format IMRaD [24] and is based on the following six sections. (1) Introduction: What is the problem being studied and why is it being studied? (2) Background: This section presents the background according to three points: first, the study of integrating individuals in workplaces, and their contextual situation; second, analysis of the methodologies and design techniques for the inclusion and/or adaptation of workplaces to the workers; and third, analysis of cyber-physical workplaces. (3) Conceptual framework: How and with what materials was the problem studied? It describes the research procedures employed. (4) Methods, techniques, and tools: What methodology was found? This section explains how the final methodology has been obtained. (5) Case study: How this methodology can be applied to a real context to obtain results. (6) Discussion: What do the results mean? (7) Conclusion: It is here that the contribution of research is reaffirmed, and new lines of research to encourage future collaboration are proposed.

## 2. Background of Literature

**Figure 1:**
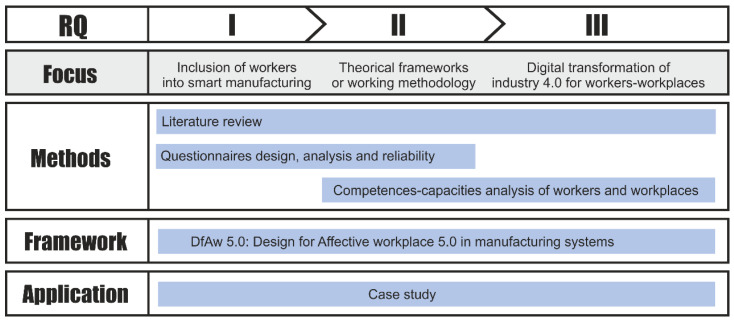
Research organization. Source: own elaboration.

Based on the types of literature review proposed by Mayer [25], a Status Quo review will be carried out for answering the research questions (RQ I–III) in the present work as showed in Figure 1.

### 2.1. Conceptual Frameworks and Design Models for Integrating the Human Factor in Smart Manufacturing System

There are numerous frameworks for accessible design. Accessibility is understood as the possibility of having access, crossing, or entering a place or activity without any limitation due to deficiency, disability, or incapacity [26]. Among the existing design types, this paper considers a design framework based on DfX, where the parameter “X” constitutes the areas of action and knowledge emerging from research to achieve the objectives. This knowledge is based on medical, psychological, or therapeutic research, and is articulated from engineering in a model for variety that is based on different theories: (1) activity theory, (2) required variety law, and (3) enactive paradigm, which are supported by the potential of intelligence and digital transformation. 

**Figure 2:**
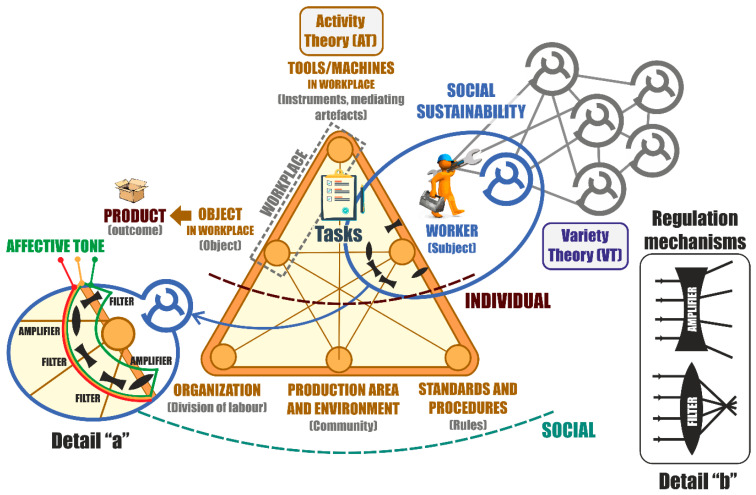
Adaptive activity system for workers. Source: own elaboration.

The theories and their applicability to the proposed model for the variety of individuals in the workplace are described below:

1. Activity theory (AT) is a widely known socio-historical approach to the analysis of value creation activities and was initiated by Lev Semyonovich Vygotsky [27,28]. Leont’ev [29] continued with this work, and Engeström expanded this knowledge. Engeström [30] was a key author in the development of AT by designing generic templates to capture data and analyze work, which have found many applications. Activity theory strives to model work and its organization. The key elements of AT are reflected in Figure 2. It distinguishes between operational activity, which integrates the subject who conducts the activity, tools, and tasks with which the worker carries out the action, and the object on which he or she performs the work. The elements of the operational activity are taken together with the context in which it takes place, and it is characterized by the community in which the action is located, the establishment of an organization, and rules of the game for the development of the operational activity.
  In the social dimension, it is possible to identify the contradictions between its elements. These contradictions, represented by the network structure, relationships, and characteristics of the social elements, are extracted from AT studies and constitute a relationship of conflicting situations of the elements of the social dimension.
2. Required Variety Law proposed by Ross Ashby states that “only the variety can absorb the variety” [31]. This determines the existence of a regulator-regulated system where the workplace takes the place of the regulator, and the users represent the regulated side of the system. The adaptation of the required variety of the system is imposed by the requirements and the definition of the workplaces. Adaptation makes possible treatment of the systems, which, in their regulatory and regulated roles, fail to present a comparable variety. The adaptation of varieties consists of reducing one part and increasing the other in such a way that the requirements of the variety law are fulfilled. A variety regulator acts in two directions: amplifying and filtering. The regulation of the relationships between the entities of the AT model increases variety in one direction (amplifier), while in the other direction it decreases variety (filter) as shown in Figure 2, Details “a” and “b”. The variety of competences-capacities, operational routines of the work, and their required organization is embedded in the model of activity theory based on the conception of variety law.
3. Enactive paradigm. The social and labor incorporation of humans involves their integration into the socio-technical framework of work systems, and starts with the assessment of their possibilities of articulating a set of competences and capacities in the workplace. New paradigms of embodied or enactive cognition appear from cognitive science [32], in which it is argued that cognition is based on and deeply limited by the nature of the body of human and established from situated knowledge, more specifically the field of manufacturing systems studies aimed at conceiving manual work under the embodied mind [33]. Enactivism stresses that the beginning of intelligence is in the body in action [34]. Its reason, according to Varela et al. [35], is based on the arrangement of the interrelation established between the body and the environment, and more specifically between the body and the mind. Thus, the human body is analyzed, not only the brain, as a source of cognition, where its movements and actions, guided by perception in the world, make much of the effort necessary to achieve the objectives [36]. The enactive theory of cognition provides a paradigm for the design of new biologically inspired cognitive architectures, with an important influence on aspects of self-organization and emerging properties [37], likewise autonomy and adaptability [38], in order to offer improvements in decision making of decisions [39]. The enactive paradigm of acting and knowing is characterized by the following elements [40]:
  - Cognition as the creation of meanings by a body agent (worker) through loops of perception-action involving a corporeal brain, located in a context.
  - Action as the coupling of the human being to the occupational environment through the body, where active cognition is an emerging form of the changing experience through loops of perception-action.
  - Embedded cognition. The agent’s mind is completely and intimately interwoven with the environment.
  - Extended cognition: A concept for the integration of the boundaries between mind, brain, body, and environment. Humans take extended cognition to increasingly distant extremes through tools and technologies.
  - Socially situated cognition. Socially situated or distributed cognition depends on the communication of ideas and emotions through sight, hearing, touch, and other sensory modalities.
  - The affective dimension. The cognitive agent constructs meaning in its context through the proposals or possibilities offered by the environment. A valuable object or context attracts, while the threatening object repels.

From the characteristics of the enactive paradigm and by using Norman’s theory of the action model [41] reconceptualized under the principles of the enactive paradigm, it is possible to characterize a worker’s demand for enactive coupling in the workplace, in general, and to allow its adaptation to the variety of a particular worker. The demands derived from the model are (1) cognitive processing, or the way a person understands work and interacts with it from the psychological perspective; (2) movement, proprioception, and contact with work in which the person tries to understand how he or she interacts with work from the physical perspective; (3) social interaction and communication, where the aim is to understand the way in which the individual relates with the rest of the individuals in the environment; (4) flexibility to change, showing the capacity to adapt to unexpected or changing situations; and (5) sensitivity to the environment and security, by being aware of the influence that the environment causes on the person.

### 2.2. Cyber-Physical Environments and Work Systems with Key Enabling Technologies (KETs)

The digital transformation is determined by technological change and the way people, in the context of the workspace, begin to interact with objects and other people, and also by the relationships between products, their connections, and their control [42]. 

Today, manufacturing plants can be monitored and controlled by means of the KETs, but this does not apply to data related to human effort. Control systems aimed at monitoring the social sustainability of factories have not been analyzed in the literature. Sustainability is a crucial topic that industries need be concerned with. The development of sustainable processes, products, or industrial services is indeed essential to ensure a respectable growth of society, which complies with new standards and guidelines. The implementation of smart sensors in manufacturing plants to understand and monitor parameters that could influence social sustainability and in the efficiency of operations is necessary [43].

The interconnection between devices and people presents a trend for the technological development of the future [44]. Cyber-physical systems serve to enable and control the activities in hybridized environments and work systems that contemplate the real and virtual part of the system with digital twins [45] associated with big data and artificial intelligence in the cloud. These technologies have also improved the quality of life for workers through remote monitoring, assistance, and vigilance. In addition, there are a wide variety of emerging technologies that allow the hybridization of the real and virtual world, such as sensors in products and processes [46], Bluetooth devices to detect parameters, wearables [47], collaborative robotics and automation [48], self-monitoring for inclusion in the work environment [49], virtual reality for employability and interviews [50], virtual training agents [51], process simulation and virtualization [52], social networks, advanced cloud computing, digital twins, and collaborative platforms. These technologies allow a symbiosis between the applications or enabling technologies in the workplace, which can be employed to assist in the development of the tasks, monitoring, and supervision of the worker in a personalized way and in the work done in real time. This constitutes a fundamental element for the regulation of jobs and workplace systems, since it integrates specific capabilities to the creation of value [53]. Other specific KETs for human effort are [54] use of xR technologies as a means of assisting with training, maintenance, and complex tasks; smart wearable solutions; using social networking services, enabled by real-time mobile communication capabilities; and using big data analytics techniques to leverage real-time information to drive the right response to avoid errors, quickly identify problems, and prompt the right decisions to improve operational efficiency among many other technologies.

## 3. Conceptual Framework

This background provides a new area of research where certain needs have been identified as requiring intervention, and opportunities for improvement and optimization can be observed, which are considered along with the questions that drive the research conducted.

The proposed framework starts with the level of establishment of workers and workplace variety to set adaptation strategies for the required variety between the two elements. This framework is articulated under a methodological approach that has been called design for affective workplaces (DfAw 5.0), which calls for the evaluation of the initial state of the system, identifies the level of demands of the workplaces, and compares them with the characteristics of workers once they have been evaluated. The Required Competences-Capacities for the Workplace (RCC) will be extracted from the evaluation, in other words, the workplace necessities and their correspondence with the Competences-Capacities regarding the profile of the worker (CCP) that has been evaluated. This proposed design model is a closed-loop model and feeds back throughout its lifecycle, oriented towards continuous improvement. In the proposed framework, it is of special relevance to articulate AT and VL in order to analyze the elements of the workplace for the establishment of variety compatibility strategies.

As regards the worker, it is necessary to ascertain his/her Competences-Capacities of the Profile (CCP) through the study of the characteristics of the subject. In this way, a generic profile can be determined, one that represents the CCP of worker. This second assessment provides information that enables the comparison of the RCC with the CCP, and therefore identifies where this work position presents weaknesses in the required variety of the worker, and the areas of competences-capacities.

**Figure 3:**
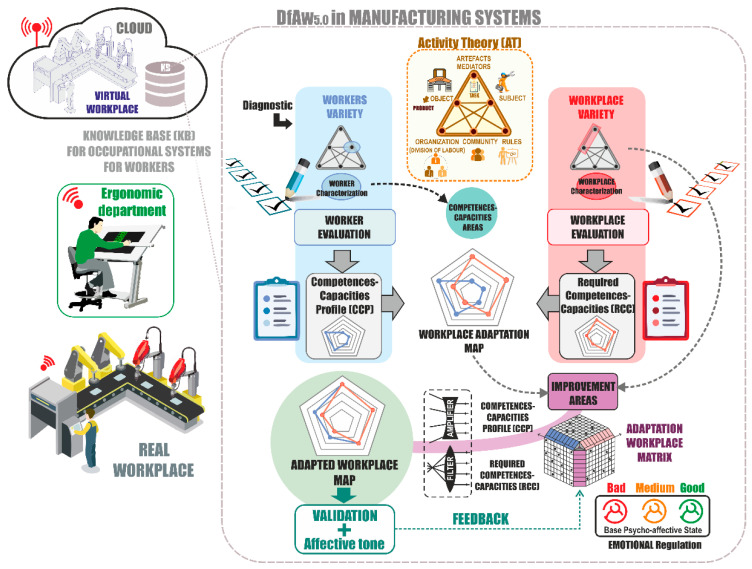
Design model for variety in a workplace for workers. Source: own elaboration.

Figure 3 shows the design model with the steps to follow until the required workplace is obtained. The use of a polar diagram is proposed for the representation and comparison of the results of the evaluations of the five established areas, since it allows for the straightforward visual identification of which Competences-Capacities areas show the most deviation. The model graphically compares the RCC to a group of defined competences-capacities areas, and contrasts these graphically by using a Workplace Adaptation Map. In the deviations of the Competences-Capacities Map, the areas to be improved are identified; these are specified by means of the articulation of variety filters and amplifiers in order to arrive at the Adapted Workplace Map, which becomes the goal of the new workplace. Together with the technical solutions on the workplace, which allow variety adaptation and make the working flow possible, the proposed model identifies the affective work experience and the emotional state of the worker by using KETs. Hence, the worker is provided with strategies of self-regulation of these emotions, under an enactive approach to the work.

The adaptability index, taken as the difference between the mean value of the five areas of the RCC assessment in the workplace and the CCP of the worker, is a parameter that has been introduced into the model to evaluate the viability of the changes. The purpose of the framework is to define an adaptability model of workplaces and industrial application systems that would benefit all the stakeholders involved, with a focus on social sustainability and workplace inclusion for workers. This concept, together with the development of methods for the evaluation of workplaces and workers, is laid out in detail in the following sections.

**Figure 4:**
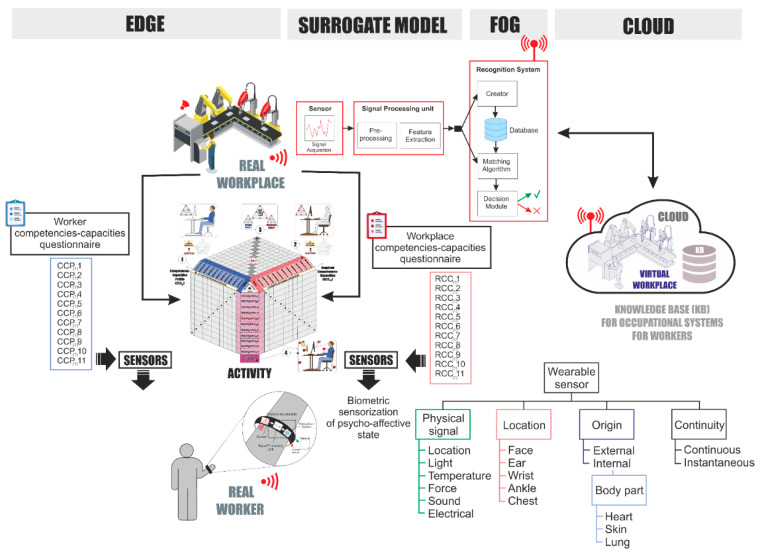
Protomodel for information processing of design parameters, capabilities, and psycho-affective state. Source: own elaboration.

Figure 4 shows how to obtain data on the different variables that characterize the work environment and the psychophysiological aspects of the worker’s state in the development of the activity, its processing, and the required feedback through a surrogate model, as well as the variety of sensors and wearables [55].

Each dimension that characterizes the enactive paradigm is defined by a set of variables that are identified through a questionnaire in the design phase and later in the work process through a set of sensors integrated in wearables, which are processed locally in real time (edge) to customize the environment for the worker [56,57] in the nearby environment (fog) and thus adjust to the worker the parameters of the surrogate model that allows the adaptation of the workplace to a particular worker through machine learning and other affective design algorithms [58]. Finally, data from sensors and wearables are sent to the cloud to update the surrogate model, adapting the work environment to the worker in an evolutionary way.

The system development requires the formulation of a framework to identify and characterize the different variables of the enactive dimensions as well as the demands of the workplace, the identification of parameters for the adaptation of the work environment, and the surrogate model that will establish the most appropriate parameters for the work environment based on the psycho-affective states between the worker and the workplace.

In the implementation and even the development of the proposed model, the worker will be assisted by one or more co-workers. 

## 4. Methods, Techniques, and Tools

The development of the model begins with (1) the analysis of variety where the worker has been previously studied for the company to find his/her suitability for the workplace. To this end, (2) each of the five different variety areas are selected to start with the predetermined questions of the questionnaire for the workers and the workplace. These questions give rise to results that are mapped on radar graphs. Once completed, (3) the results are compared between workers and the workplace to establish the appropriate design parameter, and (4) finally, when the design parameter has been selected, filters or amplifiers of variety are employed to adjust the Competences-Capacities between workers and the workplace.

**Figure 5:**
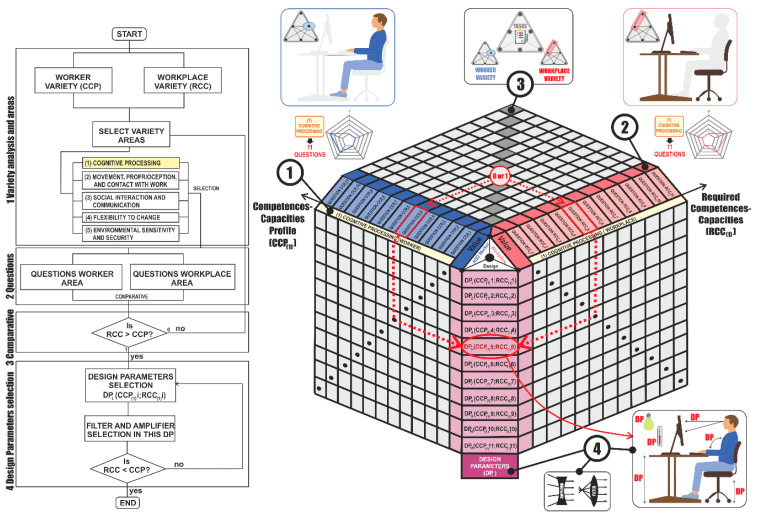
Design matrix for variety of workers. Source: own elaboration.

In this section, a particular case of analysis is presented in the variety area number one of cognitive processing with its 11 questions and the design parameters derived from questions is shown in Figure 5.

A fundamental factor for the inclusion of workers in the workplace corresponds to a specification of its initial diagnosis, which is provided to the company. The preparation of the questionnaire and the determination of its validity and reliability [59] have been carried out on the basis of a set of techniques as detailed below:

- Questionnaire design. Operationalization, understood as the process of building the instrument, consists of translating the dimensions of the worker construct into measurable elements, that is, moving from the dimensions to the indicators and from the indicators to the questions. From the characteristic features of the enactive paradigm and through the use of Norman’s theory, it is possible to characterize the worker for the purposes of the demands of a workplace. In this case, five dimensions have been established, described, and justified in Section 2.1 of the background as (1) cognitive processing; (2) movement, proprioception, and contact with work; (3) social interaction and communication; (4) flexibility to change; and (5) environmental sensibility and security. The multidimensional conception worker construct ensures that the content of the questionnaire was designed to identify the potential for the enactive coupling of workers to the workplace, by structuring it into 48 questions on the five dimensions that characterize the previous. The items are evaluated on a Likert scale from 1 to 5. The psychometric characteristics of the questionnaire were obtained through different types of statistical analyses performed with the help of the IBM SPSS Statistics program.
- Validity analysis. Validity is analyzed in terms of content and construction. Content validity of the questionnaire is carried out by an expert opinion procedure involving three psychotherapists and three occupational ergonomics technicians. There is agreement among them and hence no determination of the content validity index is required. With regard to construct validity, the orthogonality of the dimensions and the optimal number of factors or dimensions are determined by means of an exploratory factorial analysis, while maintaining the same number, that is, the five dimensions established. The KMO and significance level value of Bartlett’s test of sphericity are significant and therefore determine the validity of the instrument.
- Reliability. The reliability of the questionnaire in the different orthogonal dimensions according to the factorial analysis is ascertained by (1) an analysis of internal consistency to give meaning to the questions of the questionnaire and (2) an analysis of the discrimination capacity of the items, in order to reinforce the one-dimensional character of the test. First, Cronbach’s alpha coefficient is calculated, which is based on the average inter-element correlation and assumes that the items (measured on a Likert-type scale) measure the same construct. It is concluded that they are highly correlated, with a value of 0.785 in the questionnaire. Secondly, the Student’s t-test is used to contrast the null hypothesis that indicates the non-existence of differences between the means of the established groups, as well as indicating the homogeneity index of each item, that is, Pearson’s correlation coefficient between the score in the item and the sum of the scores in the remaining items. The reliability index of the questionnaire for the items in the different dimensions is above 0.82, a value that is considered to be good.

Since the items in the questionnaires are paired (each item on the workplace corresponds to a question regarding the workers), the design matrix for the required variety can compare the results of the two questionnaires and can identify when the demand for one (workplace) is higher than the Competences-Capacities provided by the other (worker). By comparing all the scores, a criterion is established by which the comparison of the rows in the matrix returns a “1” if it detects that the value of the response for the workplace is greater than the value of the response for the worker, or a “0” otherwise. Furthermore, this table shows which design parameters must be modified, due to any discrepancy between the demands of the workplace and the Competences-Capacities of the worker, in each collection area in the analysis of the activity of the workplace in accordance with the Workplace Description Form. These parameters are derived from the detailed study of the tasks proposed in the work form under the model of the activity theory.

The application of the filters and amplifiers in all the areas of improvement or adaptation to the workplace provides the Adapted Workplace Map, where all the vertices of the required Competences-Capacities area should be included in the competences-capacities area of the profile. Design matrix analyses for workplace variety can be carried out using a single matrix for the different Competences-Capacities areas by overlapping them in the axes (RCC) and (CCP) or with different matrices for each competence-capacity. Each of the steps in the development of the design matrix is described below.

In view of the different contexts and countries, these questionnaires need to be specified due to the fact that they are strongly influenced by operational and cultural factors at work in these contexts. The data are confidential and reserved.

### 4.1. Variety Evaluation of Workers: Competences-Capacities Profile (CCP)

The purpose of the evaluation is for the integration (sensory-motor and affective) in the workplace and the occupational therapy implied in its development. For the needs of the study, and with the purpose of ascertaining the abilities of the workers and the difficulties found in his or her interaction with the job, a questionnaire has been designed to address the workers and their supervisors. The questionnaire is composed of 48 sentences classified in accordance with the five areas of Competences-Capacities as defined in the research section about the enactive Paradigm, and with relevance in the occupational activity. For the codification of the items of this questionnaire, the acronym CCP(i)k will be used, where “CCP” corresponds with the “Competences-Capacities Profile”; the sub-index “i” corresponds with each of the five classification areas; and “k” is the question number. Each line of the questionnaire is scored according to a Likert scale from 1 to 5, where “1” represents total agreement with the question, and “5” total disagreement. The questions are formulated in order to ensure that an affirmative answer implies the lowest demand for the user. 

From the evaluation of the group of questions in each area, the Competences-Capacities Profile index is extracted for each defined area and calculated as the mathematical mean of the evaluations carried out by the different members of the design or redesign team for the workplace.

Several of the questions are of a generic character, but a large number are oriented towards the requirements of a workplace in an industrial plant. The aim is to focus the analysis on the objectives and to make the questionnaire easier to fill in by excluding questions that are of no practical application in this specific study. 

**Figure 6:**
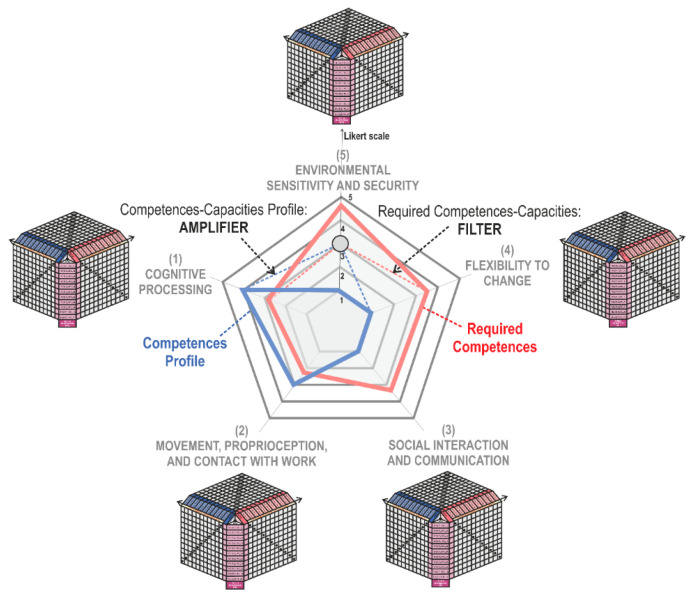
Map for competences-capacities profile (CCP), required competences-capacities (RCC), and filters and amplifiers effects on an adapted workplace map. Source: own elaboration.

The whole profile is represented in a polar graphic or Competences-Capacities Profile Map as shown in Figure 6.

The list of items that comprise the questionnaire is grouped into the five competences-capacities areas based on the worker as detailed below and which comes from the study of the enactive paradigm:

1. Cognitive processing. The worker’s competence and capacity are evaluated with reference to aspects such as planning, decision-making, understanding verbal or written instructions, and the recognition of elements within the work environment.
2. Movement, proprioception, and contact with work. This area analyzes the worker’s competences-capacities that can influence the tasks and physical activities required in the workplace, and all aspects of manual work and direct contact with the product. The use of tools and contact with materials, as well as movements and safety risks present in the workplace, must also be considered in this competence area.
3. Social interaction and communication. This area assesses the worker’s competences and capacities in the social environment and his/her response to interaction with colleagues or supervisors.
4. Flexibility to change. This area assesses the worker’s difficulties in adapting to change and his/her response to changes in the work routine.
5. Environmental sensitivity and security. This area evaluates the response of the worker to the characteristics of the environment where the workplace is located. It includes the reaction to the presence of noise from outside the workplace, moving elements, and changes in the areas around the workplace, etc. Safety questions, such as the worker’s responses to emergency situations, are also considered in this section.

The questions in each of the areas are shown in Table 1. 

### 4.2. Workplace Variety Evaluation: Required Competences-Capacities (RCC)

A workplace can be defined as the space occupied by a person in an organization to achieve production specifications in a defined area by developing the tasks that are integrated within its activity. This space is integrated by the physical area that the worker occupies, as well as the operational area, determined by the set of physical and mental tasks that the worker must perform during the working day, and with the responsibilities associated with the activity that characterizes the workplace.

In addition to focusing on workers, the physical and social environments around the workplace and health and safety measures are also considered. The ones last-mentioned are variables that have an indirect influence on work performance. 

After analysis, using the activity theory model, and obtaining the variety of the characteristics that define a workplace, we can say the variety will be a function of the following concepts: (1) required tasks and activities, (2) responsibilities, (3) tools and other support, (4) required qualifications, (5) environment, and (6) product to be produced.

Studying the activity model of the job under these six perspectives, it is possible to characterize different workplaces. The Workplace Description Form should be prepared by a person who knows the details of the workplace in the company, together with the members of the workplace adaptation design team. 

At this point, the characteristics of the workplace are known, but there is no way to relate them effectively to the characteristics of the workers. For this purpose, the approach is to develop a questionnaire that allows the evaluation of the workplace, according to the five areas of Competences-Capacities identified for workers. The questions are addressed to the design and ergonomics department of the company. 

As well as the Competences-Capacities Profile (CCP) questionnaire of workers, the Required Competences-Capacities (RCC) by workplace evaluations include 48 questions organized into the five previously defined areas. Each item of this questionnaire corresponds to an item of the profile questionnaire of workers. In this way, it can be established that both questionnaires allow the correlation of the same concepts, allowing the members of the design team to compare them. For the coding of the questionnaire, the acronym RCC(j)z will be used, where “RCC” corresponds with the “Required Competences-Capacities”, the subscript “j” is one of the five classification areas, and “z” is the question number. Similarly, questionnaire responses are based on a Likert scale from 1 to 5, where 1 represents complete agreement with the question and 5 represents complete disagreement. Questions are also formulated so that an affirmative response will determine the lowest demand in the assessed Competences-Capacities area for the workplace. In this way, the higher scores will give an idea of where one should act to make the job adequate to the competences-capacities of this special population. 

When all responses to the different items have been collected, the Required Competences-Capacities Index (RCC) can then be calculated as the arithmetic mean of the ratings for each of the competence areas. These are represented in a polar graph defined as the Required Competences-Capacities Map in Figure 6.

Table 2 summarizes the proposed questions for the evaluation of the workplaces. The proposed questionnaire, although it is focused on the same areas of Competences-Capacities, differs in accordance with the nature of the workplaces analyzed.

### 4.3. Adapted Workplace Map and Interpretation

After the evaluation of the users and the workplace, the next step involves the analysis of the results. For this purpose, we propose building the Adapted Workplace Map: a graphic representation that compares the results of the two questionnaires. As shown in Figure 6, two maps are built on the same graph: The Competences-Capacities Profile Map and the Required Competences-Capacities Map regarding the workplace necessities. 

The Adapted Map represents how adapted the workplace has become to the characteristics of the population or those of a specific subject that is to be incorporated into the work. By translating this assessment into the polar graph, it is possible to state that the more common the area is in the polygons represented on the map, the higher the demand becomes for competence required by the workplace. 

Another way of analyzing and evaluating the aforementioned adaptability is by means of the adaptability index as the difference between the mean value of the five areas of RCC evaluation of the workplace and the CCP of the workers; this parameter has been introduced into the model to evaluate the viability of the changes.

Figure 6 shows that the required competences of the workplace have a very high demand in the area of the competences related to sensitivity to the environment, although their demand in terms of movement, proprioception, and contact with the work is very low. When compared with the profile of the worker’s competences, it can be observed that, in the area of sensitivity to the environment, the required competence of the workplace is too high for the worker to fulfil. However, this could be adapted without difficulty in terms of cognitive processing or movement, since certain competences of the worker exceed those which are required in the workplace. This means that the vertices of the Competences-Capacities Profile that are within the area of the Required Competences-Capacities determine the areas of the workplace that must be adapted to the population. Adjusting the workplace requires the use of variety regulators in two directions. On the one hand, this involves the modification of elements of the workplace to reduce its demand (filters) or the adoption of solutions that increase the worker’s Competences-Capacities (amplifiers). This resource can be employed to assess the degree of adaptability of existing jobs to specific workers.

### 4.4. Variety Filters and Amplifiers

Once the worker’s profile of competences and capacities has been established, then the job requirements and the degree of coincidence have to be evaluated and determined. It is necessary to establish adaptation mechanisms through Design Parameters (DPs), structured on variety filters and amplifiers. The filters and amplifiers are linked to the relations of the worker with the other elements included in the activity model, and these are shown in Figure 6.

Variety filters and amplifiers are concepts that incorporate Ashby’s Variety Law to reduce or increase the variety of the components of an interaction system. The filters and amplifiers can be defined as follows: (1) variety filters associated with design changes that are oriented towards reducing the demands of the workplace, that is, reducing the required competences-capacities; and (2) variety amplifiers, which are all changes that can be adopted to increase the competence of a worker to operate within a particular workplace. 

All the variety filters and amplifiers resulting from the study of the characteristics of workers, the occupational environment, and the work system including KETs are compiled in Table 3. For their establishment, the elements and relationships of activity theory have been considered, that is, the five areas of Competences-Capacities defined from the difficulties in sensory integration, and the perception of the environment by the workers.

The design parameters are not exclusive; they can contain other parameters depending on the type of sector or on the workplace. 

As indicated in previous sections, this study corresponds to the subjective experience of the worker during a working day as a consequence of the operational activity. These affective aspects have been integrated into the proposed methodology design as shown in the following section.

### 4.5. Implementation of Proposed Model for Self-Regulation Embodied in the Workplace. Execution Matrix of Embodied Emotion in Workers 

Despite the existing literature on regulatory strategies, the act of bringing such interventions widely into mainstream settings remains an open challenge. Workers may experience difficulty with regulating their emotions in the workplace due to a deficiency in processing stimulations and setting them in context, and, as a result, their environment can cause a high degree of stress and discomfort [60]. This determines the need to provide the person with affective self-regulation of the experience in the workplace, in order to develop productive activity [61]. 

Embodiment theories predict that activating conceptual knowledge about emotions can be accompanied by re-experiencing bodily states, since simulations of sensory, motor, and introspective experiences form the foundation of conceptual representations of emotion [62]. The emotions and affects resulting from experience in the workplace determine processes in the organism known as appraisal and arousal, which can be evaluated by psycho-physical variables through wearables and communicated via interfaces to the worker. In this way, these processes can be self-regulated by the worker or controlled by the cloud information system through subrogated models.

**Figure 7:**
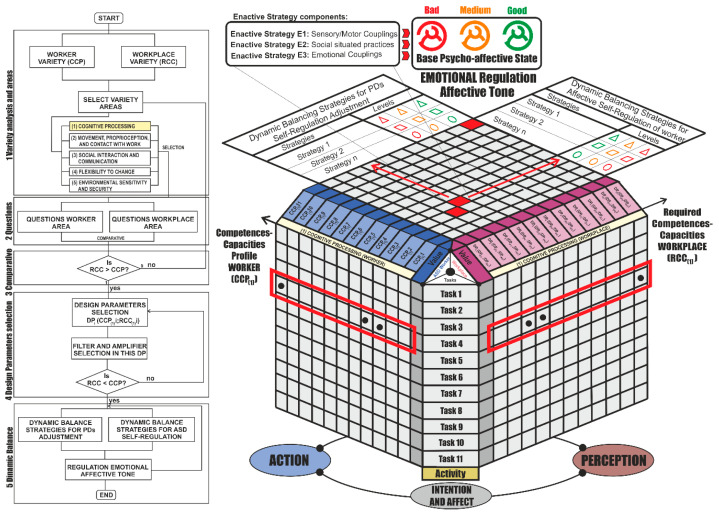
Execution matrix for cognitive variety under Norman’s enactive model. Source: own elaboration.

In order to articulate the competences of subjects with the demands of the workplace, two sets of dynamic balance strategies are articulated: (1) strategy of affective dynamic balance with the possibility of articulation by means of self-regulation by workers, which determines a psycho-affective state of the active flow, and (2) strategy oriented towards operational autonomy with corporal coupling and sensory-facilitated experience, including social relations, as shown in Figure 7.

Based on the analysis established in Figure 7, the enactive conception of the labor activity highlights the idea that the origin of intelligence is in the body in action, and underlines the nature of cognition: to be in action [34]. Its argument is founded on the fundamental premise, first raised by Varela et al. [35]: “perception and cognition depend essentially on the interactions of the organism with its environment. In other words, perception and cognition are limited and influenced by the conditions of the embodiment of that cognitive body agent”, which is particularly true for workers.

The strategies of co-regulation are established in the phases of interaction and operational intervention included in Table 4. The principles for their creation and possible modification have been adapted from [63], and are set out below:

- Principle 1. Co- and self-regulation are structured in the form of stepwise navigation depending on worker diagnosis.
- Principle 2. A limited set of messages (in visual code, emoticons, etc.) is shown regarding simplification, predictability of behavior, and clarity.
- Principle 3. Co- and self-regulation strategies are trained in the home and school environment. Indo-democratic strategies are included.
- Principle 4. Information media oriented towards the visual channel are used, under analogies of an intensity-type thermometer (analogical) that enables the intensity of the emotion and the strategy of confrontation adapted to the intensity to be selected.
- Principle 5. The level of emotional intensity communicated must be oriented towards the promotion of the selection of a coping strategy, and not towards the communication of the type of emotion.
- Principle 6. The effect of the content of the media that embodies the strategy that takes shape has to coincide with the level of intensity of the emotions.
- Principle 7. A tailored self-regulatory and co-regulatory tool should be designed for workers with different conditions who share the same functional needs.

Assuming the aforementioned principles, and under the consideration of the potentialities that come from the incorporation of KETs and IoT platforms with artificial intelligence incorporation and learning or deep learning machines, the strategies of Table 4 have been formulated for their implementation with industry 4.0 technologies.

This approach enables a dynamic balance of the affective states that determine the realization of productive activities adapted to the competences of the workers with online support and the articulation of filters and amplifiers for the workplace.

**Figure 8:**
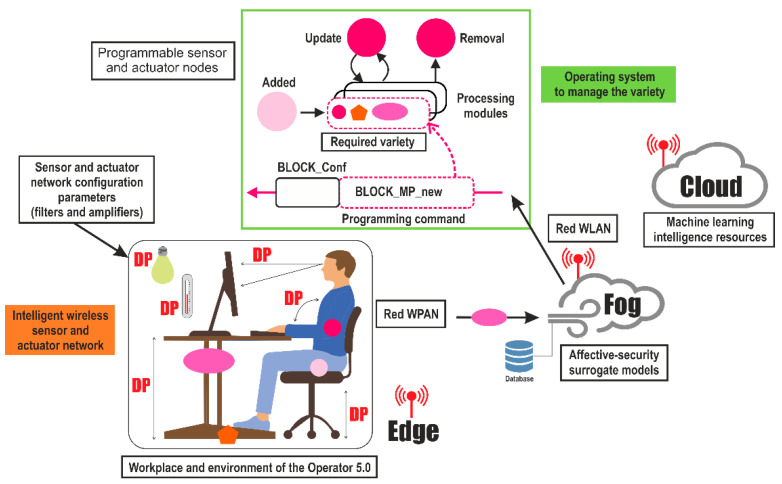
Wireless sensor network to provide affective and safety support to the operator 5.0 Source: own elaboration.

The Figure 8 shows how to integrate the values of the workplace parameters (filters and variety amplifiers) and the affective state of the worker through a network of intelligent wireless sensors. These sensors take the information from the edge to send it to the fog, where the management and optimization of the operational parameters of the subrogated models include not only affective aspects in real time, but also safety aspects.

The design of the system enables its flexibility and adaptability depending on the characteristics of the worker occupying the workplace and the manufacturing process to be carried out. For this, as shown in the figure, it is necessary to activate or deactivate sensors and parameters of adjustment of workplaces, as well as to obtain from the cloud the subrogated model associated with the operational conditions of the master production plan.

The implementation of the system can be carried out with a WPAN network architecture that connects to the WLAN network of the industrial plant, under the criteria established for sensors by the IEEE standard for WBAN body sensor networks IEEE 802.15.6. [64].

## 5. Case Study

The proposed conceptual framework establishes a method for designing inclusive workplaces. In order to implement the proposal, this section presents the analysis and evaluation of workers with autism spectrum disorder (ASD) and workplaces in an industrial plant dedicated to the manufacture of metallic parts. According to the Diagnostic and Statistical Manual of Mental Disorders, autism spectrum disorder (ASD) is a neurodevelopmental disorder characterized by impairments in social interaction/communication and by restricted and repetitive behavior (American Psychiatric Association [65]). The motor system also plays a role in the pathophysiology of ASD [66], which determines different types of therapeutic needs, and makes it complex to generate multidimensional design solutions that can be adapted to all members of the public involved [67] including in the design of workplaces. The manifestation of ASD involves the presence of difficulties that affect everyday life from an early age and continue through adulthood; these difficulties are problematical to eliminate [68,69]. This situation makes it complicated to propose specific solutions and to carry out research on the integration of individuals with ASD into the community and into occupational environments of vital importance throughout their lifecycle.

The majority of research conducted on products and environments that are adapted to individuals with ASD have focused on children and on intervention therapies using products that improve the heterogeneous group of autistic symptoms [70]. Various research studies into therapies and techniques for the improvement of social behavior have been considered, such us (1) products present in daily life [71]; (2) toys with several different parts and actions that include adult participation by incorporating sound effects [72]; (3) products to accomplish daily routines and activities [73]; (4) products that enable cooperative actions to be imitated [74]; (5) objects that constitute a circuit of activities and that include multisensory stimulation [75]; (6) objects to develop aquatic activities [76]; and (7) virtual reality [77], digitalization, virtual stimulation, robots and humanoids, tablets, digital systems, and information and communication technologies (ICTs), among many others [78,79]. Previous work indicates that there is potential for research into the configuration and implementation of work systems and occupational environments in the adult lifecycle phase of individuals with ASD [80].

**Figure 9:**
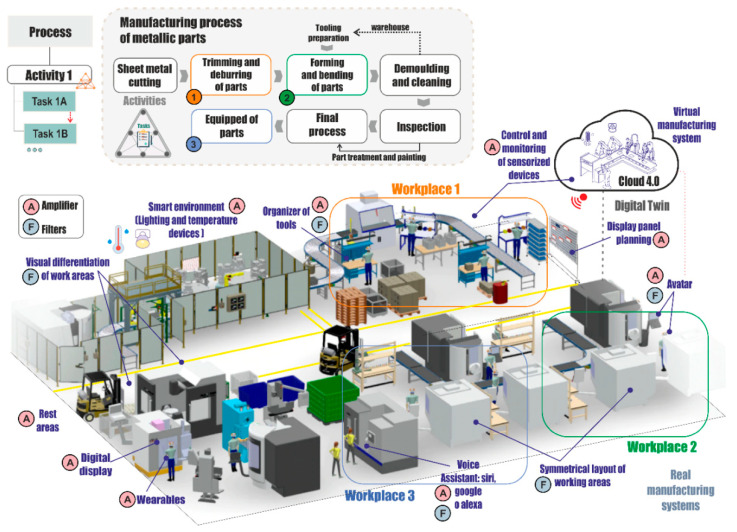
Embodied emotion in individual with ASD with filters and amplifiers. Source: own elaboration.

In this case study, the production process consists of several workplaces of which three will be analyzed, as shown in Figure 9. 

The process flow can be observed with the three workplaces analyzed. The production flow starts with the cutting of the raw material. The process is performed by an automated machine that cuts the patterns with the help of a program that optimizes the use of the material. The operator in this phase takes the finished sheets that come out of the machine and organizes them into kits for each route map associated to the different parts. These kits are supplied to the forming workplace where the mold is prepared, the surface is cleaned, and the release agent is applied. The sheet is then placed on the tool and the part is formed. Parts that have been shaped are verified and undergo the final processing stage for treatment and painting. Finally, the parts that have been checked in the final processes are equipped with sealants and rivets.

The main phase for the application of the design model for variety effectively includes ascertaining how individuals with ASD perceive the environment around them and, consequently, the way they interact with the environment and the competences they can develop in the five dimensions, which we have characterized from the active paradigm. To this end, all the workstations in the organization that can potentially be occupied by the ASD worker are analyzed. The results of the questionnaire are showed in Table 5, for each of the competence areas. 

Having assessed the Competences-Capacities of workers with ASD and the workplaces, the adaptation of the workplaces can be established. For this purpose, the suitability of filters and amplifiers of variety must be analyzed. Derived from relating the questions between the ASD worker and the workplace, the types of design parameters (DPs) are shown in Table 6.

Once the filters and amplifiers have been selected, each is regulated by means of digital facilitators that provide the workplace with intelligent technologies and establish the external technological support for the worker in the workplace. 

The adaptation map for workplace 3 is shown in Table 7. The demand for the workplace (red line) is located above the worker’s Competences-Capacities profile (blue line), but the deviation is small. This implies that, with small modifications in all areas, a good adaptation of the workplace could be achieved, although a greater effort will be required to adapt the working environment in order to minimize the demand.

The Competences-Capacities profile area is completely contained within the required Competences-Capacities polygon. Filters and amplifiers have been set up in all the competence areas, and notable changes have been observed as shown by the dotted lines with respect to the adaptation map of the workplace.

In the proposed model, the embodied or enactive perspective and the concept of dynamic balance that provides support and assistance for the regulation of the emotion enable the design of workplaces and more efficiently integrate the person into the world of employment through affective resonance. The proposed design of the occupational environment and its adaptive self-regulation require an information system, supported by machine learning and Rough set techniques in the cloud, which establish dynamic balance strategies oriented towards the modification of the DPs of the workplace, and strategies for affective self-regulation under the enactive conception (embodied mind) of the worker with ASD.

The establishment of the variety filters and amplifiers that constitute the design parameters, as showed in Figure 9 and Table 6, together with the strategies of dynamic affective and emotional balance have been conceived from the enactive paradigm. The use of avatars is proposed for the emotional self-regulation of workers with ASD. Self-regulation from the cloud is employed to deliver the affective self-regulatory elements to the ASD and the design parameter levels. The design parameters include collaborative and therapeutic robots, with capacities for social interaction and with support for training, virtual reality, and proposals for affective activities and therapies, which replace the operator support of co-workers and supervisors assigned to the person with ASD under the concept of work with human assistance.

**Figure 10:**
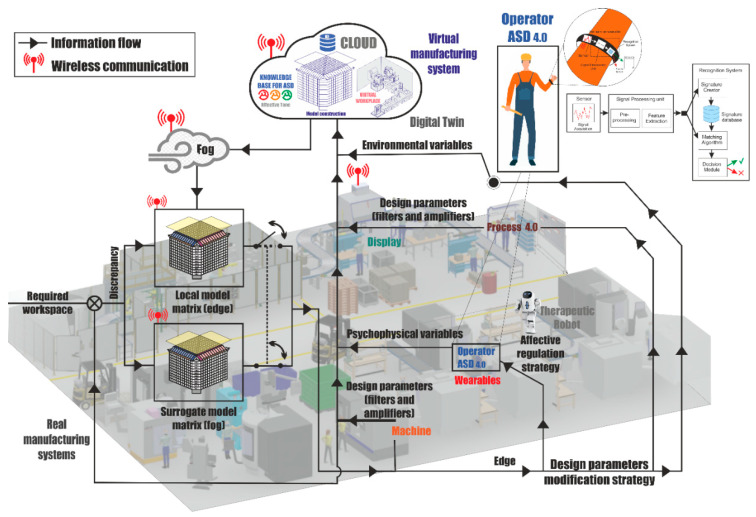
Enactive workspace interface for workers 5.0. Source: own elaboration.

Architecture and information flow of the enactive workspace interface for workers is shown in Figure 10. This architecture contains the model of dynamic regulation of the effects of the ASD worker and of the design parameters (DPs) of the work system, to guarantee the enactive coupling between the ASD worker and the workplace system in the development of the task.

It comprises three main parts: (1) the boundary or field (edge) from which the environmental variables of the productive elements are extracted, as well as the psycho-physical elements of the worker with ASD, which are sent to the cloud from the edge. From the edge, a local operation mode, which enables the self-regulation of the CCP and RCC, is possible by modifying the pre-established dynamic balance strategies from the local model; (2) the cloud, or the place where the edge data arrives, which are processed with big-data, machine learning, and Rough Set techniques, among others, make up the dynamic balance model, for the self-regulation of the work environment, thereby generating a new subrogate model that is better adapted to the individual with ASD that will be sent to the cloud periodically; and (3) the fog that contains the surrogate model for the self-regulation with affective dynamic balance strategies of adjustment of DPs, from the cloud, which will be used for the control and self-regulation of the active coupling of the ASD worker with the workplace system.

Self-regulation from the fog is employed to send the affective self-regulatory elements to the ASD worker and the design parameter levels. As shown in Figure 10, the design parameters include collaborative and therapeutic robots, with capacities for social interaction as well as support for training, virtual reality, and proposals for affective activities and therapies, which are replacing the operator support of co-workers and supervisors assigned to the person with ASD under the concept of work with human assistance.

**Figure 11:**
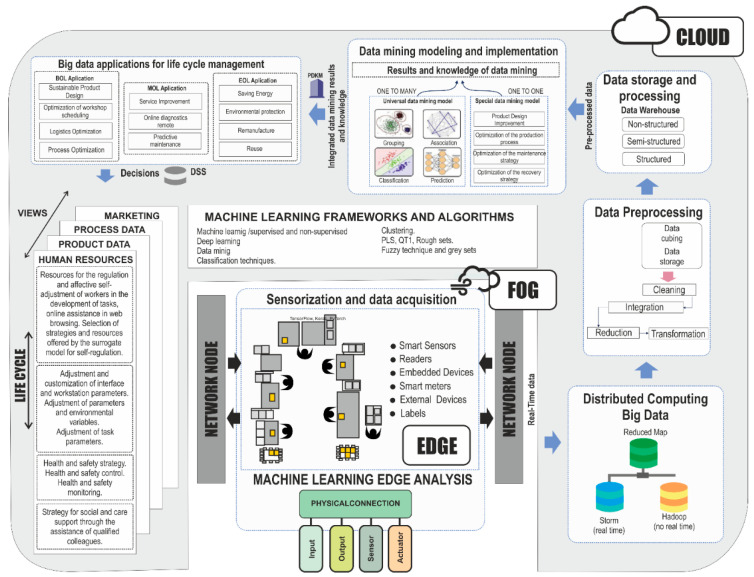
Affective system data architecture based on surrogate models. Source: own elaboration.

Figure 11 adapted from [81] includes for each of the life cycle phases, the necessary stages that constitute the informational view of the model.

Firstly, this includes sensorization and data acquisition at the edge, followed by processing in fog, massive data ingestion, and its subsequent storage and treatment under cognitive computing. Secondly, once the data capture and storage process is completed, this information is used in the realization, improvement and updating of the surrogate models that attend to the different areas and levels of the production system. Finally, these models and their application information are visualized and managed by specific applications, in the different stages of the life cycle, departments, or views, through indicators in integrated dashboards, among others.

The creation, implementation, and exploitation of surrogate models supports the worker’s affective systems by using equipment and industrial systems located on the edge to acquire data and measurements in real time. For data acquisition, intelligent cognitive applications and algorithms are embedded to adapt and mediate interactions between physical environments. The presence of the digital twin in the fog or cloud has the ability to optimize the parameters of the surrogated models and manage data ingestion into the cloud. The cloud platform generates the surrogate models and updates them, soul-stringing them and sending them to the fog for execution.

Figure 11 and Table 8 cover the various frameworks and algorithms in cloud, fog, and edge that have been collected from [56,57,58,82].

## 6. Discussion

In the analysis of the possible reasons for the need for inclusive employment in industries and services, one of the main problems identified involves the lack of frameworks, methodologies, and tools to develop solutions whose results ensure real and efficient adaptation to the development of productive activities by workers in occupational environments. 

Hitherto, the design methodologies studied have been oriented towards products and environments, due to the detection of the opportunity to offer a solution that allows companies to integrate a sustainable design model and to incorporate their talent [83] for the inclusion of the labor offered by workers. The design framework considered is based on three theories such us activity theory, variety law, and the enactive paradigm. This theories offer separate advantages, such as organization and help in decision-making from activity theory, regulation and dinamization from variety law, and the suggestion of modeling the worker as a cognitive agent on the basis of body-mediated sensory–motor interactions with the environment based in the enactivism approach [84]. A special interest is linked to the associated Competences-Capacities, which can be classified as cognitive, referring to the capacity of the human brain; physical, related to the muscular capacity; and mechatronic mechanisms or systems. Their combined action allows shows the potential of this proposed model. 

The research regarding AT is substantial and includes many contributions in the fields of: learning in collaborative environments [85], online learning [86], digital teaching [87], collection and analysis of learning data [88], creativity development methodology [89], information systems [90], apps [91], inclusive education [92], and Building Information Modeling (BIM) implementation [93]. Since the factors that characterize a workplace can be static and dynamic [94] and not all of these factors are open to change, AT proposes that, in the analysis of human activities and especially in the occupational environments, it is necessary to articulate mechanisms to regulate the variety. From this stance, this theory focuses on the design and characterization of the different subjects and develops tasks in the workplaces in a qualitative way to provide an approach to a template that facilitates the analysis of the information gathered from the different nodes as well as the study of possible contradictions. Activity theory has not yet allowed an analysis of the work for the cognitive, psychomotor, or sensory variety of the subjects that carry out the activity, nor does it incorporate variety regulators (filters and variety amplifiers) for the relationships of the subject with the other elements, and it therefore lacks an instrument that modulates, analyzes, and synthesizes the variety of execution that occurs in special populations such as those including ASD adults.

A crucial topic discussed in this paper involves how the human body, not only the brain, is understood as the source of cognition, given that the body, its movements, and its actions, guided by its perception of the environment, make most of the effort needed to obtain the objectives [36]. This is contrary to traditional ideas of how the mind works, characterized by the so-called computational metaphor of the mind, where cognition is considered as the manipulation of symbols of internal mental representations, designed to produce a requested result/behavior [95], and where the body is reduced to an input/output device. 

Finally, in order to manage the smart adaptation of the worker to the workplace environment, and for him or her to execute the work in an adapted way, there are KETs that provide inclusion and regulation of the worker within the workplace [96]. Cyber-physical systems have also been employed not only to promote better results for both employees and employers, but also for the improvement of behavior and creativity in the workspace. These technologies have also improved the quality of life and general public health in gerontological and diseased populations through remote monitoring, assistance, and vigilance. Another key concept involves the virtual avatars. The Virtual World environments that have been created involve people (as avatars) in spaces that simulate the real world in the form of “places where the imaginary meets the real”. A more comprehensive definition is that a Virtual World is “a synchronous, persistent network of people, represented as avatars, facilitated by networked computers” [97,98,99]. Workers with ASD are represented by avatars and can talk to each other by voice or text chat, in public or in private. This facilitates their interaction with other workers. In the workplace, all these technologies allow a symbiosis between the applications or enabling technologies that can be utilized, in a personalized way and in real time, to assist in the development of the tasks and in the monitoring and management of the worker and the work done. This constitutes a fundamental element for the regulation of jobs and workplace systems for the individuals with ASD, by integrating their specific capabilities into the creation of value [53].

## 7. Conclusions 

This paper establishes a general methodological framework for workplaces of workers, called DfAw 5.0, which enables the situation of an organization to be ascertained in relation to the adaptability of their workplaces to the characteristics of those individuals who could become members of their workforce. This model is characterized by supporting four principles: (1) In the participatory or co-design process, family members are incorporated to transfer their knowledge and experience, along with colleagues who provide support in the workplace and other HR agents of the company; (2) design principles in the solutions demanded by the job are collected, such as simplicity, predictability, clarity, and emotional support; (3) the potential of KETs and the digitalization of industry 4.0 are incorporated in order to conceive an inclusive strategy in the workplace; (4) the progressive autonomy of workers in the workplace is acquired in phases of immersion referring to observation, guided intervention or imitation with performance supports, and emotional support of colleagues involved in the process of adaptation of the workplace. The autonomous operation phase is carried out with operational assistance and affective self-regulation through ICT. 

Workplace transformation characteristics with requirements that are far from the competences and capacities of workers vary widely, and their needs can differ considerably from one to another. A conceptual model has been established in the methodological proposal for the employment inclusion of workers, which promotes the development of life independence, and for them to be considered as an active part of society in all stages of their lifecycle. This responds to the possibility of establishing a model of worker inclusion according to the RQ I.

According to RQ II, the absence of generic theoretical frameworks to assist in the selection of functional requirements and design parameters of workplaces and to encompass the wide variety of worker needs has led to the establishment of a framework from three researched theories, activity theory, law of variety, and enactive paradigm, in order to cover the complexity of signs and behaviors of socio-technical systems and the modern digital world.

In order to articulate the digital transformation potential of Industry 4.0 and an innovative model based on new conceptual frameworks required in RQ III, the digital tools are used within the methodological framework to make the workplace more dynamic, and to establish the monitoring and control of the work environment designed for the variety of workers. In addition, the proposal constitutes an active information architecture based on avatars, which allows the dynamic regulation of emotions together with the loops of sensory-motor coupling and social relations that workers with ASD develop. Consequently, any emotional state or workflow of a task in the worker’s activity can be regulated by means of dynamic balance strategies. This application can be used by organizations through the use of current tools, such as machine learning, rough set techniques, and Big Data. 

Due to the wide variety of people, the surveys developed are based on a broad profile that considers the range of difficulties associated with these workers. In this case, the focus has been on a job in an industrial sector, such as the manufacturing of metallic parts. However, by making minor modifications to the questionnaire, it can be adapted to other sectors, and the model could be equally applicable. As future work, design aspects can be specified, such as methods and times, and can be studied with a more ergonomic approach, as well as studies of movements or routes in the plant.

It is important to highlight that there is a perceived cognitive workload for “smart operators” and the new requirements of the digital factory. A study on the latest models developed to assess fatigue and psychological stress over time due to the “new” tasks required of smart factories would provide a complete state-of-the-art scenario in relation to the interaction of smart operators in the smart manufacturing system. As a future development, it is necessary to investigate aspects such as: Model of Human Error Probability based on dual-phase approach for learning process in cognitive-oriented tasks [100], Healthy Operator 4.0: A Human Cyber-Physical System Architecture for Smart Workplaces [101], and Heart rate variability based assessment of cognitive workload in smart operators [102].

Currently, work is being carried out at the informational level with the data from the digital enablers and their indicators in order to detect, collect, and measure the real-time information of the standard workers and workers with special needs in the workplace together with the analysis of the information using the digital enablers.

Finally, as an open line of research in the long-term, it is proposed to ascertain whether these techniques are suitable for their exportation to other disorders and contexts.

## Figures and Tables

**Table 1:** Collect data through questions for the evaluation of the Competences-Capacities profile (CCP). Source: own elaboration.

Key n°	Questions
(1) Cognitive processing	
CCP_(1)_1	The worker has difficulty planning a sequence of tasks
CCP_(1)_2	The worker has difficulty making decisions regarding the task being performed (outside of work instruction)
CCP_(1)_3	The worker has difficulty understanding instructions and executing them
CCP_(1)_4	The worker has difficulty finding objects in the environment
CCP_(1)_5	The worker has difficulty recognizing objects that are distributed arbitrarily
CCP_(1)_6	The worker has difficulty understanding numbers and symbols
CCP_(1)_7	The worker has difficulty following the instructions of other people
CCP_(1)_8	The worker has difficulty expressing his/her needs
CCP_(1)_9	The worker has difficulty in agreeing with other people
CCP_(1)_10	The worker has difficulty identifying the defects caused (mistakes in execution).
CCP_(1)_11	The worker experiences difficulty in identifying the mistakes in execution
(2) Movement, proprioception, and contact with work	
CCP_(2)_12	The worker presents spasmodic and repetitive movements
CCP_(2)_13	The worker finds it difficult to manipulate small objects
CCP_(2)_14	The worker presents hypersensitivity to forced postures
CCP_(2)_15	The worker has difficulty using tools
CCP_(2)_16	The worker is easily disoriented
CCP_(2)_17	The worker has apparent difficulty in keeping his/her balance
CCP_(2)_18	The worker tends to lean on nearby objects
CCP_(2)_19	The worker is hyposensitive to pain or heat
CCP_(2)_20	The worker does not tolerate strong smells
CCP_(2)_21	The worker tends to taste unknown substances
CCP_(2)_22	The worker avoids contact with particular materials/textures
CCP_(2)_23	The worker loses his/her balance if he walks with objects in his/her hand
(3) Social interaction and communication	
CCP_(3)_24	The worker has difficulty reacting when appointed
CCP_(3)_25	The worker has difficulty interacting with strangers
CCP_(3)_26	The worker tries to avoid interaction with other people
CCP_(3)_27	The worker responds in a negative way to the proximity of persons who are not part of the environment
CCP_(3)_28	The worker shows signs of stress when exposed to other people (invasion of personal or proxemic space)
CCP_(3)_29	The worker responds negatively to physical contact with other people
CCP_(3)_30	The worker presents symptoms of stress when feeling judged
(4) Flexibility to change	
CCP_(4)_31	The worker has difficulty adapting to improvised changes
CCP_(4)_32	The worker has difficulty adapting to changes in the established routine
CCP_(4)_33	The worker has difficulty changing tasks
CCP_(4)_34	The worker subscribes to routines and protocols.
(5) Environmental sensitivity and safety	
CCP_(5)_35	The worker is distracted by the surrounding noise
CCP_(5)_36	The worker responds negatively to unusual sounds in the environment (e.g., occasional use of machines, unloading trucks, etc.)
CCP_(5)_37	The worker is alarmed by unexpected sounds
CCP_(5)_38	The worker is slow to react to alarm sounds
CCP_(5)_39	The worker expresses discomfort at bright lights
CCP_(5)_40	Worker is distracted by warning lights (e.g., flashing beacon on a wheelbarrow)
CCP_(5)_41	The worker expresses discomfort at bright colors
CCP_(5)_42	The worker is shocked by the movement of objects in the environment (e.g., movement of the bridge crane)
CCP_(5)_43	The worker becomes disoriented if objects in the environment are moved
CCP_(5)_44	The worker has a high tolerance for pain and does not react immediately object is hurting him
CCP_(5)_45	The worker may have compulsive movements that may cause injuries to himself or to people in the environment
CCP_(5)_46	The worker has difficulty interpreting warning indicators and relating them to the actual danger
CCP_(5)_47	The worker manifests fatigue after performing tasks involving repeated movements (e.g., sanding)
CCP_(5)_48	The worker needs to escape temporarily to avoid sensory overload

**Table 2:** Collect data through questions for the evaluation of the required Competences-Capacities (RCC). Source: own elaboration.

Key n°	Questions
(1) Cognitive processing	
RCC_(1)_1	The workplace does not require planning and organization of activities by the worker
RCC_(1)_2	The workplace does not require decision-making on activities (outside of work instruction)
RCC_(1)_3	The documentation presents simple and perfectly sequenced instructions
RCC_(1)_4	Documentation is clearly identified and located
RCC_(1)_5	The tools have a defined location and can be easily sorted
RCC_(1)_6	The workplace does not require written information (forms, records, etc.)
RCC_(1)_7	The supervisor’s verbal instructions are clear and concise
RCC_(1)_8	The workplace does not require interaction with the supervisor
RCC_(1)_9	The workplace is independent of other workers
RCC_(1)_10	A bad execution of the work can be detected/corrected without consequences on the final product
RCC_(1)_11	Visual aids are used to execute certain tasks (e.g., projections)
(2) Movement, proprioception, and contact with work	
RCC_(2)_12	The workplace does not require manual precision
RCC_(2)_13	The workplace does not require precision tools
RCC_(2)_14	The workplace is ergonomic (height, etc.)
RCC_(2)_15	The tools to be used are ergonomic and easy to use
RCC_(2)_16	The job does not require moving to other production areas
RCC_(2)_17	The workplace does not require moving with severe safety risks
RCC_(2)_18	The worktables, trolleys, and elements that make up the post are robust and stable
RCC_(2)_19	The workplace does not expose the worker to irritating substances
RCC_(2)_20	The workplace does not expose the worker to substances with a strong odor
RCC_(2)_21	The workplace does not expose the worker to toxic substances
RCC_(2)_22	The workplace does not involve contact with viscous substances or dust
RCC_(2)_23	The workplace does not require moving objects with hands
(3) Social interaction and communication	
RCC_(3)_24	The workplace does not require supervision (high autonomy)
RCC_(3)_25	The workplace does not require rotation of personnel
RCC_(3)_26	The workplace does not require constant interaction with co-workers
RCC_(3)_27	The workplace is visible to other staff
RCC_(3)_28	The workplace is not located in a confined space
RCC_(3)_29	The workplace does not require proximity to other colleagues, not even sporadic physical contact
RCC_(3)_30	The activities are not subject to severe inspections with the possibility of rejection (trial)
(4) Flexibility to change	
RCC_(4)_31	The work is fully planned at the beginning of the shift
RCC_(4)_32	Work is planned in the medium term (e.g., weekly)
RCC_(4)_33	The nature of the work varies frequently, in form or cadence (not constant)
RCC_(4)_34	The work is routine and repetitive
(5) Environmental sensitivity and security	
RCC_(5)_35	The environment of the workplace is quiet and free from background noise (machinery, hammering, tapping, etc.)
RCC_(5)_36	No exceptional external noises (e.g., occasional use of machines, unloading trucks, etc.) are usually produced in the workplace environment
RCC_(5)_37	There are no loud audible signals around the station (e.g., door open warning)
RCC_(5)_38	The security system (e.g., fire alarm) has other means than the audible alarm to transmit the alert
RCC_(5)_39	There are no constant light signals (flashing, projections, etc.) in the vicinity of the station
RCC_(5)_40	The lighting of the station is adequate, there are no dazzling light bulbs or flickering lights (fluorescent)
RCC_(5)_41	In the workplace environment, the colors are neutral and unobtrusive
RCC_(5)_42	In the workplace environment, there are objects that are not part of the work in progress (conveyor belt, overhead crane, etc.)
RCC_(5)_43	The environment of the station is fixed and always maintains the same configuration (there are no elements that can change place)
RCC_(5)_44	There are elements in the surroundings of the post that can be harmful (edges, corners, etc.)
RCC_(5)_45	There are either no tools in the vicinity of the station that could cause injury or, if there are, they are kept under supervision
RCC_(5)_46	There are no moving elements in the surroundings of the station that could lead to entrapment
RCC_(5)_47	Around the post there are benches that allow for occasional rest
RCC_(5)_48	There are rest areas around the post

**Table 3:** General design parameters: filtering (DP_F_) and amplifying (DP_A_). Workplace improvement areas. Source: own elaboration.

FILTERS (DPiF)	AMPLIFIERS (DPiA)
Lighting (DP1)	
DP1(F1): Place windows and skylights that increase the availability of natural light. DP1(F2): Replace fluorescent lamps with LEDs, which also reduces energy consumption by 60%. DP1(F3): Use more points of light distributed over the working area by reducing the intensity of each point. DP1(F4): Avoid glossy finishes that may cause glare on furniture surfaces, walls, or floors.	DP1(A1): Place curtains or blinds so that light passes through but still prevents distractions. DP1(A2): Use dimmers to adjust the lighting conditions to the needs of the worker. DP1(A3): Place crystals that attenuate the incidence of the sun’s rays inside the room.
Color Usage (DP2)	
DP2(F1): Use neutral colors in the workplace. DP2(F2): Avoid elements with bright colors, especially if they are large. DP2(F3): Use colors in harmony with the rest of the elements. DP2(F4): Use color to highlight positive space.	DP2(A1): Use bright colors on visual devices.
Workspace organization (DP3)	
DP3(F1): Delimit areas of activity. DP3(F2): Use contrast in divisions or windows. DP3(F3): Organize the elements of the work area symmetrically. DP3(F4): Use repetitive patterns in the arrangement of objects. DP3(F5): Encourage linearity. DP3(F6): Avoid dispersion of the elements, group them together to form a weighty entity.	DP3(A1): Avoid large, open spaces. DP3(A2): Place organizers that facilitate order and avoid having objects in sight (by using drawers, cupboards, etc.).
Environmental Noise (DP4)	
DP4(F1): Fit acoustic panels to ceilings and walls. DP4(F2): Use sound-absorbing floors. DP4(F3): Isolate work areas from noise. DP4(F4): Set up a piped music system that plays sounds that improve concentration.	DP4(A1): Wear headphones that protect against noise and allow music to be heard.
Manual Contact (DP5)	
DP5(F1): Use soft finishes. DP5(F2): Avoid surfaces that are rough or unpleasant to touch.	DP5(A1): Wear soft gloves.
Temperature (DP6)	
DP6(F1): Maintain control of temperature and humidity levels.	DP6(A1): Keep temperature low, never above 22 °C.
Contact between people (DP7)	
DP7(F1): Develop large work areas that respect personal space. DP7(F2): Widen passages to prevent unwanted clashes.	DP7(A1): Maintain fixed templates in work areas. DP7(A2): Minimize staff turnover. DP7(A3): Employ specialized worker supervisors (training/recruitment programs).
Clothing and Individual Protection Equipment (IPE) (DP8)	
	DP8(A1): Provide cotton work clothes. DP8(A2): Provide long-sleeved uniforms. DP8(A3): Provide comfortable, lightweight safety shoes. DP8(A4): Provide lightweight, non-tightening glasses and masks.
Delimitation (DP9)	
DP9(F1): Dimension spaces with partitions, furniture, and floor markings. DP9(F2): Use color contrasts between the floor and other surrounding elements (wall, partitions, furniture, etc.).	DP9(A1): Develop individual jobs.
Signaling (DP10)	
DP10(F1): Use route marking on the floor. DP10(F2): Differentiate passage work zones by clearly marking the contours. DP10(F3): On stairs, mark the contour of the steps with bright colors.	DP10(A1): Use stairs with wide treads and handrails on both sides.
Layout (DP11)	
DP11(F1): Define short and direct passageways between sections. DP11(F2): Arrange related work areas in a contiguous manner.	
Furniture (DP12)	
DP12(F1): Use appropriately proportioned furniture (ergonomic). DP12(F2): Use stable and robust furniture. DP12(F3): Use safety elements in drawers and doors to prevent trapping. DP12(F4): Avoid furniture with parts that protrude from the general volume (wheels, legs, etc.). DP12(F5): Use self-braking wheels.	DP12(A1): Place organizers that facilitate order and avoid having objects in sight (by using drawers, cupboards, etc.). DP12(A2): Arrange positions where it is possible to work in a seated position. DP12(A3): Use height-adjustable seats. DP12(A4): Use height and tilt adjustable tables.
Instructions (DP13)	
DP13(F1): Provide panels in front of the working areas as a visual aid. DP13(F2): Provide simple, easy-to-understand graphic instructions. DP13(F3): Employ stepwise sequencing.	DP13(A1): Provide specialized training programs. DP13(A2): Provide specialist worker supervisors (training/recruitment programs). DP13(A3): Encourage working with support. Include warning devices for the supervisor such as light beacons.
Documentation (DP14)	
DP14(F1): Migrate paper documentation to digital systems. DP14(F2): Employ stepwise sequencing.	
Key Enabling Technologies, KETs (DP15)	
Sensors; Wearables; Robotics; Interactive whiteboards; Self-monitoring; Artificial vision; Artificial intelligence; Virtual reality; Augmented reality; Simulation and virtualization; Learning machines; Biometric devices; Temporal and spatial models; Virtual Avatars.	
Training, coaching and workplace support (DP16)	
Workers; Supervisor; Tutor; Technological operator; Collaborative robot; Education and training programs.	

**Table 4:** Self-regulatory strategies embodied (enactive) in the design of the workplace. Source: own elaboration.

Occupational Interaction		Occupational Context	Intervention
Objective: Sensor-motor coupling in the workplace	Objective: Social practice located in the workplace	Objective: Emotional attachment to the job and work environment	Types and levels of intervention in self-regulation
Operational Feedback Strategies in the workplace	Strategies for Social Interaction in the workplace	Strategies with emotional content	The steps for interaction are: Step 1. Identification of emotions by facial recognition, biometric wearables, etc. Step 2. Emotional correction by the support partner or self-regulation by the workers. Levels of Intervention: Relaxation methods. Self-regulation strategies with photos. Self-regulation strategies with videos. Rest strategy of the workers.
Explicit representations in visual language preferred. Message with an analogical one-dimensional signal (shape) that changes. Empty signals (as content receivers) in which meaning emerges through interaction in the workplace and working environment.	Impose social practice in accordance with established standards. Create new social practices in accordance with the new rules. Use and strengthen existing social practices in a way through which a whole new meaning of interaction emerges.	Measurement, prediction, and prevention with predefined solutions by behavioral models. Predefine support of co-regulation and self-regulation with different options. Empty support strategies (as content receivers) that are formed in accordance with the welfare objective.	

**Table 5:** Competences-Capacities profile of worker with ASD and required Competences-Capacities of the workplace. Source: own elaboration.

Competences-Capacities Areas (C-C Areas)	Evaluation *					
	Worker with ASD	Workplace 1	Worker with ASD	Workplace 2	Worker with ASD	Workplace 3
(1) Cognitive Processing	1.6	4.1	2	1.7	1.6	2.1
(2) Movement, Proprioception, and Contact with Work	1.6	2.1	1.8	2.8	1.6	2.2
(3) Social Interaction and Communication	1.3	3.1	1.3	1.7	1.3	1.4
(4) Flexibility to change	1.3	3.8	1.8	1.5	1.3	1.5
(5) Environmental sensitivity and safety	1.8	2.4	1.9	3.3	1.8	3.1

* The evaluation values of the worker with ASD and the workplace are calculated as the average results of each area. For example, the values marked in **blue** and **red** represent, respectively, the result of the evaluation of the worker and the workplace. The value of 1.6 in the area of cognitive processing is the average of the answers to the 11 questions: Average (1,2,2,1,1,3,1,2,1,2,2) = 1.6.

**Table 6:** Adaptation of Competences-Capacities profile of worker with ASD and required Competences-Capacities of the workplace. Source: own elaboration.

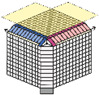				CCP_(1)_1	CCP_(1)_2	CCP_(1)_3	CCP_(1)_4	CCP_(1)_5	CCP_(1)_6	CCP_(1)_7	CCP_(1)_8	CCP_(1)_9	CCP_(1)_10	CCP_(1)_11	
				Worker Evaluation (1.6) *											
Key	DPs			1	2	2	1	1	3	1	2	1	2	2	
RCC _(1)_1	Scheduling	Workplace Evaluation (**2.1**)*	2	1	X	X	X	X	X	X	X	X	X	X	Filter
RCC _(1)_2	Instructions		2	X	0	X	X	X	X	X	X	X	X	X	
RCC _(1)_3	Sequencing		2	X	X	0	X	X	X	X	X	X	X	X	
RCC _(1)_4	Space organization		4	X	X	X	1	X	X	X	X	X	X	X	Amplifier
RCC _(1)_5	Space organization		3	X	X	X	X	1	X	X	X	X	X	X	Amplifier
RCC _(1)_6	Documentation		2	X	X	X	X	X	0	X	X	X	X	X	
RCC _(1)_7	Instructions		2	X	X	X	X	X	X	1	X	X	X	X	Amplifier
RCC _(1)_8	Instructions		1	X	X	X	X	X	X	X	0	X	X	X	
RCC _(1)_9	Contact with people		1	X	X	X	X	X	X	X	X	0	X	X	
RCC _(1)_10	Instructions		1	X	X	X	X	X	X	X	X	X	0	X	
RCC _(1)_11	Signaling		3	X	X	X	X	X	X	X	X	X	X	1	Filter

* Values in brackets represent: worker (**blue**) and workplace (**red**) mean values.

**Table 7:** Adaptation of workplace 3. Source: own elaboration.

C-C. Areas	Evaluation		Adaptation		Adapted Competences-Capacities Map
	Worker with ASD	Workplace 3	Worker with ASD	Workplace 3	
(1)	1.6	2.1	2	1.5	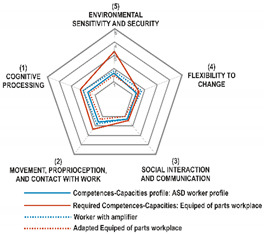
				
(2)	1.6	2.2	1.8	1.3	
				
(3)	1.3	1.4	1.3	1	
				
(4)	1.3	1.5	1.8	1.5	
				
(5)	1.8	3.1	1.9	1.6	
				

**Table 8:** Machine learning frameworks and algoritms for fog, edge, and cloud for affective cyber-physical systems with sensorized surrogate models. Source: own elaboration.

Objective	Description	Layer	Machine Learning Frameworks
Affective-Rational Objective	Construction of surrogate models for the long-term management and control of work environment parameters based on the variety filters and the activity developed by the worker.	Cloud	Machine learning/supervised and non-supervised Deep learning Data mining Classification techniques. Clustering. PLS, QT1, Rough sets. Fuzzy technique and grey sets
Affective instinctive objective	Adjustment of parameters and variety filters in the surrogate model in real time and short term.	Fog	Machine learning. Meta heuristics and mathematical optimization algorithms. Evolutionary optimization algorithms. Genetic algorithms. Analytical optimization techniques.
Rational Affective Instinctive Objective	Personalization and self-adjustment of parameters of the worker-subrogated model, supervised by machine learning.	Edge	Supervised machine learning. Cooperative algorithms based on game theory.

## Data Availability

Not applicable.
